# Common Genetic Variants Link the Abnormalities in the Gut-Brain Axis in Prematurity and Autism

**DOI:** 10.1007/s12311-018-0970-1

**Published:** 2018-08-14

**Authors:** Elżbieta M. Sajdel-Sulkowska, Monika Makowska-Zubrycka, Katarzyna Czarzasta, Kaja Kasarello, Vishal Aggarwal, Michał Bialy, Ewa Szczepanska-Sadowska, Agnieszka Cudnoch-Jedrzejewska

**Affiliations:** 1 Department of Experimental and Clinical Physiology, Center for Preclinical Research, Medical University of Warsaw, Warsaw, Poland; 2 Department of Psychiatry Harvard Medical School and Brigham and Women’s Hospital, Boston, MA 02115 USA

**Keywords:** Preterm birth, Microbiota-gut-brain axis, “Leaky gut”, Vagus nerve, Angiotensin receptor type 2

## Abstract

This review considers a link between prematurity and autism by comparing symptoms, physiological abnormalities, and behavior. It focuses on the bidirectional signaling between the microbiota and the brain, here defined as the microbiota-gut-vagus-heart-brain (MGVHB) axis and its systemic disruption accompanying altered neurodevelopment. Data derived from clinical and animal studies document increased prevalence of gastrointestinal, cardiovascular, cognitive, and behavioral symptoms in both premature and autistic children and suggest an incomplete maturation of the gut-blood barrier resulting in a “leaky gut,” dysbiosis, abnormalities in vagal regulation of the heart, altered development of specific brain regions, and behavior. Furthermore, this review posits the hypothesis that common genetic variants link the abnormalities in the MGVHB axis in premature and autistic pathologies. This hypothesis is based on the recently identified common genetic variants: early B cell factor 1 (EBF1), selenocysteine tRNA-specific eukaryotic elongation factor (EEFSEC), and angiotensin II receptor type 2 (AGTR2), in the maternal and infant DNA samples, associated with risk of preterm birth and independently implicated in a risk of autism. We predict that the AGTR2 variants involved in the brain maturation and oxytocin-arginine-vasopressin (OXT-AVP) pathways, related to social behavior, will contribute to our understanding of the link between prematurity and autism paving a way to new therapies.

## Introduction

This review considers an association between prematurity and autism supported by epidemiological data. It focusses on the disruption of the gut-brain communication and presents a new hypothesis of the common genetic link between prematurity and autism.

The rise in global prematurity rates has been accompanied by an increase in prevalence of significant neurodevelopmental disorders including autism spectrum disorders (ASD) [1]; many premature infants are at higher risk of autism. According to World Health Organization [2], more than 1 in 10 infants are born premature, before 37 weeks of pregnancy, with majority born between 32 and 37 weeks, and 5% born extremely premature, before 28 weeks [3]. The median global prevalence rate of ASD, updated in 2017 was over 1 in 160 cases [4]. Among the very premature infants, around 30% develop ASD symptoms as compared to 1% in the full-term controls [5, 6]. Boys are more likely to be born prematurely and fare worse than premature girls [7]. Autistic boys not only outnumber the girls by four to one but also show more severe repetitive/restricted behaviors than girls [8]. Ex-preterm infants that subsequently develop autism show several characteristics that include lower birth weight, lower gestational age, male gender, chorioamnionitis, acute intrapartum hemorrhage, and abnormal magnetic resonance imaging (MRI) reflecting differences in brain structure [6].

Both genetic and environmental factors are implicated in preterm birth and autism; more than a few hundred genes associated with risk of autism have been identified, but the first genes associated with preterm birth risk were only recently reported [9]. And while there is a growing list of factors that increase the risk of prematurity alone [10], or autism alone [11, 12] the maternal stress, depression, and antidepressant use during pregnancy are associated with increased risk of both prematurity [13, 14] and autism [15, 16].

This review focusses on the bidirectional gut-brain communication, here defined as the microbiota-gut-vagal-heart-brain (MGVHB) axis. It examines a disruption of the MGVHB axis as a possible link between prematurity and autism by comparing symptoms, physiological abnormalities, and behavior in premature and autistic children. Indeed, clinical data have provided critical observations of increased prevalence of the gastrointestinal (GI) and cognitive problems and behavioral delays in the premature [17, 18] and autistic children [19, 20]. While animal studies have shown altered development and behavior in the microbiota-free mice [21–23], that has led to increasing recognition of the role of gut microbiota in the neuropsychiatric disorders [24].

The MGVHB axis encompasses the microbiota, the vagus nerve, sympathetic and parasympathetic pathways of the autonomic nervous system including the enteric nervous system, the neuroendocrine and neuroimmune system including the HPA axis, and the brain; more recently, the gut-vagal-heart-brain has been recognized [25, 26]. The MGVHB axis and its functions in prematurity and autism are examined here in terms of the microbiota, GI system, vagal, cardiovascular (CVS), and the central nervous system (CNS).

Additionally, we present a new hypothesis that common genetic variants link the abnormalities in the MGVHB axis in premature and autistic pathologies. These common gene variants including early B cell factor 1 (*EBF1*), selenocysteine tRNA-specific eukaryotic elongation factor (*EEFSEC*), and angiotensin II receptor type 2 (*AGTR2*), associated with preterm birth were recently identified in maternal and infant DNA samples using genome-wide association analyses on 43,568 European-ancestry females and confirmed in three birth data sets from Nordic countries [9]. They were also indexed by the online database of genes associated with the risk of autism [27].

Future studies should aim at identification of additional genes associated with prematurity as well as the reexamination of the autism susceptibility genes in the context of their function in the MGVHB axis, communication in premature infants, and neurodevelopmental pathologies, including autism. We tentatively predict that sequence variations in *AGTR2* gene, and associated abnormalities in the oxytocin-arginine-vasopressin (OXT-AVP) pathways, involved in major physiological processes and social behavior, may play an important role in the further understanding of the link between prematurity and autism paving a way to new therapies.

### Methodology

This article reviews the clinical and preclinical literature addressing the postulated association between prematurity and autism at the level of the microbiota, the gut, the vagus nerve, the heart and the brain and behavior, and in the context of newly identified gene variants associated with the risk of prematurity. Literature searches were performed using PubMed base; both original and review articles were selected by best match and the highest number of citations. Over 200 hundred papers were reviewed, out of which 140 were selected for discussion; over 80% of the reviewed papers were published within past 10 years.

### Gut Abnormalities in Prematurity and Autism

Gastrointestinal symptoms are often observed in both premature and autistic infants. Higher prevalence of sepsis and inflammatory bowel disease (IBD) and necrotizing enterocolitis (NEC) has been observed in prematurity [28], with up to 12% of premature infants affected by NEC [29]. The prevalence of GI symptoms in ASD ranges between 23 and 70% [30] including constipation, diarrhea, abdominal pain, gastroesophageal reflux, vomiting, IBD, and NEC [12, 28, 31], suggesting incomplete gut maturation and increased gut permeability, so-called leaky gut [19, 20, 32].

Normally developing human gut fully permeable in the fetus forms mucosal tight junctions towards the end of pregnancy and becomes less permeable shortly after birth in a process known as “gut closure.” In the premature neonate, the underdeveloped gut lining disrupts initial “gut closure” and the first bacterial colonization, which in turn affect the subsequent gut development [33] and the second “gut closure.” The timing of gut closure is critical for the GIT’s maturation as it regulates the integrity of gut microbiota and protects against environmental insults, including bacterial translocation.

Furthermore, in term infants, the developing gut-associated lymphoid tissues (GALT) increasingly differentiate between commensal bacteria or food-derived antigens and the pathogens [34]. In preterm infants, abnormal immune responses to commensal bacteria eventually lead to the disrupted gut epithelium and NEC [35]. Interaction of abnormal microbiota with underdeveloped GALT interferes with the pro- and anti-inflammatory balance, resulting in an inadequate immune response that disrupts normal gut development.

Importantly, there is evidence pointing to incomplete gut closure in autism resulting in increased gut permeability—the “leaky gut” [20, 32], which is the salient feature in autism [36, 37]. Increased gut permeability has also been reported in premature infants, especially those receiving formula versus milk [38].

### Altered Gut Microbiota in Prematurity and ASD

GI symptoms, observed in both premature and autistic infants, are closely related to abnormal gut microbiota characterized by a decrease in biodiversity and beneficial bacterial species [20, 32, 39]. This relationship has been supported by recent trials with probiotic therapy showing an improved microbiota colonization in preterm infants [40] and 80% reduction of GI symptoms as well as a significant improvement in behavior in autism [41].

The gut is presumed by some [42] being sterile at birth. The first bacterial gut colonization in the term naturally delivered infants includes facultative and anaerobic bacteria, *Lactobacillus* and *Bifidobacterium*, derived from mother’s vagina and feces. This first gut colonization is disrupted in premature infants [33], especially those delivered by C-section when the gut is first colonized by predominantly skin bacterial species. It was proposed that the recent increase in C-sections may contribute to the reduced diversity of gut microbiota, the “microbial deprivation syndrome,” resulting in the abnormal immune system and CNS maturation [43]. Furthermore, many of the premature infants are formula fed and on antibiotic therapy, thereby interfering with normal microbiota development.

The second gut colonization occurs around the time of weaning when the digestive system adjusts to the switch from mother’s milk to solids, and the gut is colonized by strict anaerobes. In the preterm infants, abnormalities in age-specific microflora may interfere with the maturation of epithelial gut-blood barrier and alter gut microbiota interplay [39] resulting in “leaky gut,” allowing antigen penetration, evoking the immune response [44, 45], and dysbiosis [39], and account for the higher prevalence of sepsis, IBD, and NEC [28]. A similar disruption of gut microbiota takes place in autism with a shift from beneficial bacteria to spore-producing, antibiotic-resistant, neurotoxin-producing bacteria [20, 32] and is associated with the GI symptoms^,^ and increased prevalence of IBD [28]. Animal studies suggest that changes in gut microbiota are communicated to the brain [46]. Thus, abnormalities in gut development and the “leaky gut” may contribute to neurodevelopmental abnormalities in autism [20].

### Vagus Nerve Abnormalities in Prematurity and Autism

Gastrointestinal, cardiovascular, and psychiatric disorders [47] in both premature and autistic children are presumed to be related to vagus nerve abnormalities. Increased prevalence of IBD observed in both prematurity and autism [31] reflects low vagal nerve activity (VNA) and VNA is a predictive marker of NEC [48]. Cardiovascular abnormalities observed in both premature infants [48] and children with autism [49] are also related to lower VNA. Both IBD and neuropsychiatric disorders have been shown to respond to vagal nerve stimulation [47].

Vagus nerve is a primary communication channel between the gut microbiome and the brain and is the integral component of the MGVHB axis. It regulates metabolic homeostasis by controlling visceral functions as well as the innate immune response [47, 50] and mediates the information between microbiota and brain affecting behavior [25, 47].

VNA, measured in terms of heart rate variability HRV [51], is used to estimate vagal regulation of the heart and the GI system. VNA is a function of the gestational age at birth [52] and is correlated with infant age [53].

Impaired VNA innervation may be responsible for decreased cardiac baroreflex’s (BRS) control of heart rate in premature compared to term infants [54]. Abnormalities in neuro- and socioemotional development observed in premature and autistic children may also reflect lower VNA; estimates of VNA have been used as a marker of infant development [51, 52, 55].

Vagal activity is regulated by gut microbiota. This regulation is mediated through the gut-synthesized factors [56] and increases brain synthesis of brain-derived neurotrophic factor (BDNF) [20]. Lower BDNF levels have been observed in the umbilical cord blood of premature infants than in full-term infants [57]. BDNF levels are also altered in autism [20].

### Heart Activity in Prematurity and Autism

Both prematurity and autism are associated with higher risk of cardiac abnormalities, such as heart failure in prematurity [58] and abnormal cardiac activity in autism [59, 60].

One method used to help diagnose cardiovascular disease (myocardial infarction, congestive heart failure, coronary artery disease) is the HRV, which reflects the autonomic heart regulation and the balance between the parasympathetic and sympathetic input. Preterm neonates show a less complex pattern of HRV [61] as compared to term infants suggesting a dysfunction in the autonomic heart regulation reflecting lower parasympathetic input. Importantly, a decrease in HRV [62] and the increase in heart rate have been observed in children with autism [49]. It has been suggested that autism is associated with cardiac-linked parasympathetic underconnectivity [63] reflected in lower HRV. However, no differences were detected either in systolic or diastolic blood pressure in those children [63], underlying the role of parasympathetic dysregulation in abnormal cardiovascular function in children with autism [64].

The occurrence of patent ductus arteriosus (PDA) is increased in the preterm infants [65] resulting in a decreased blood perfusion of the brain [66] that may contribute to brain injury [67] and periventricular white matter abnormalities also observed in autism [68], although PDA is rarely found in autism. The occurrence of brain injury in PDA affected infants supports the concept of the heart-brain axis and a relationship between cerebrovascular disease and cardiovascular system dysregulation [26]. Accordingly, brain regions such as the prefrontal cortex, anterior cingulate cortex, insula, hypothalamus, amygdala, and solitary nucleus regulate heart rate by the vagus nerve [69]. Interestingly, examination of postmortem brain samples derived from children with autism showed structural abnormalities in the brain structures involved in the parasympathetic regulation of HRV and tonic electrodermal activity [70]. On the other hand, the observation of altered HRV in premature infants at risk of developing NEC [71] supports the concept of the gut-heart axis and a relationship between the composition of gut bacteria and the coronary heart disease [72].

### Brain Region-Specific Changes in Prematurity and Autism

Cognitive and behavioral impairments are commonly manifested in both prematurity [73] and autism [74], with the core deficit in social behavior in autism [75], also observed in prematurity [6]. Premature infants [76] are at risk of specific learning deficits, hyperactivity, and autism [76]. Less common are motor deficits in premature [73] and autistic children [77].

MRIs in premature infants subsequently diagnosed with autism have shown reduced volume of temporal, occipital, insular, and limbic regions that are typically involved in autistic pathology [78, 79]. It has been suggested that early overgrowth in the regions mentioned above, as observed in autism, may be a compensatory response to the initial delayed growth in the premature infants [78]. Furthermore, results of the MRI brain studies suggest a relationship between preterm birth, abnormal brain wiring, and the time of onset of autistic symptoms [80], implying that brain outside the womb develops along different trajectories. Specifically, the thalamus and the thalamocortical connectivity that develop rapidly before birth play the crucial role in the development and maturation of numerous sensorimotor, cognitive, and attentional circuits [81]. Disrupted by preterm birth, cerebral maturation may thus lead to abnormalities in thalamocortical connectivity. Functional MRI studies in extremely premature infants support this notion, showing increased functional connectivity between the thalamus and lateral primary sensory cortex but reduced connectivity between thalamus and cortex in the prefrontal, insular, and anterior cingulate regions that are involved in executive, integrative, and cognitive functions [82]. Abnormal thalamocortical connectivity has also been reported in children with ASD [81], suggesting that the social and communication abnormalities may be associated with thalamic abnormalities. Anatomical and functional underconnectivity with prefrontal, parieto-occipital, motor, somatosensory, and temporal areas has also been found. On the other hand, the functional connectivity between the thalamus and the right temporal lobe was tentatively increased [81].

Brain injury is common in preterm infants and includes intraventricular hemorrhage (IVH), cerebral white matter injury (WMI), and both cortical and deep gray matter involvement [83, 84]. MRI abnormalities in ex-preterm infants that subsequently develop autism also include periventricular leukomalacia (PVL) and cerebellar hemorrhagic injury [6]. Importantly, there is a threefold increase in autism as a result of neonatal IVH and an increase in autism in girls early affected by IVH [85].

The cerebellum has emerged as a brain region involved in cognitive, executive, social, and emotional regulation in addition to its importance in motor learning [86]. Cerebellum is a key brain structure involved in autistic pathology; it is also a vulnerable structure in prematurity due to its protracted development extending to the postnatal period. The cerebellar injury is higher in the very preterm than in term infants [87]. Furthermore, there is a significant association between isolated damage to the premature cerebellum and subsequent impairment of regional volumetric growth in the contralateral cerebrum [88, 89] that may play a significant role in the development of autism. Crucially, cerebellar abnormalities have been extensively reported in autism [20, 32] suggesting an association between specific cerebellar injury in preterm infants and social behavioral deficits observed in autism [90]. The damage to the vermis replicates core autism behavior [91]; vermis is decreased in both premature [73] and autistic infants [92]. Reduction in Purkinje cell number is one of the most consistent observations in autism [93]. The altered functional activity of Purkinje cells has been implicated in both prematurity [73] and autistic-like behavior [94].

Additionally, behavioral abnormalities in prematurity and autism may be directly related to an “emotion-processing network.” Clinical data suggest that the extent of social immaturity in adolescents born prematurely was related to gray matter changes in the fusiform gyrus and indirectly the orbitofrontal region, suggesting a link between preterm birth and social impairment [95]. Importantly, disruptions in functional connectivity have been reported in the left hemisphere in fetal brain in premature infants [96] and autism [97], and abnormal neuronal dysfunctions in the left amygdala and orbitofrontal cortex in autism [32] may be related to altered social behavior [32].

### A Genetic Link Between Prematurity and Autism?

The data cited above support the notion that prematurity and autism share abnormalities in MGVHB axis that may be linked to common factors regulating fetal development. This concept is supported by two recent studies. First, functional fMRI data have shown diminished functional connectivity in the left hemisphere of the fetal brain, which precedes the preterm birth [96], suggesting that the brain changes in prematurity occur early during pregnancy rather than being triggered by the preterm birth. Second, the identification of genetic variants associated with length of pregnancy and the preterm birth [9] suggests genetic bases of prematurity. Three out of six recently identified genes, *EBF1*, *EEFSEC*, and *AGTR2*, are associated with gestational duration and preterm birth. Additional three genes, wingless-type MMTV integration site family member 4 (*WNT4*), adenylyl cyclase type 5 (*ADCY5*), and an oncogene (*RAP2C*), are associated with the gestational duration but not preterm birth. Lastly, our search for these genes in databases resulted in locating them among genes associated with increased risk of autism [27]. A succinct review of these gene functions and possible association with the MGVHB axis is presented below.

*EBF1* gene plays an important early differentiation-promoting role of several neuronal cell types including Purkinje cells [98]. Altered differentiation of Purkinje cells could contribute to the decreased Purkinje cell number and/or activity implicated in both the prematurity and autism. Reduction in Purkinje cell number that is one of the most consistent observations in autism [93] has also been observed in premature infants [73]. Cerebellar dysfunction in Purkinje cells has been implicated in autistic-like behavior [94]. Reduction in Purkinje cell size in autism has also been reported [99]. *EBF1* is involved in regulation of cell differentiation in the murine striatum [100]; mutation in this gene could likely contribute to the striatal abnormalities observed in autism [101].

*EBF1* gene plays a crucial role in early development of the gut’s B cells, and thus immunoglobulins IgM and IgG, as evidenced by an absence of B cells in *EBF1* knockout (KO) mice [102]. Alterations in immune cells, including B cells, are often reported in premature infants [103] and autism [104].

*EBF1* has also been shown to be involved in cardiac ventricular conduction system (VCS) as demonstrated by slow VCS in the *EBF1* KO mouse [105]. *EBF1* gene is involved in the control of blood pressure and carotid artery intima-media thickness. *EBF1* variants could lead to bradycardia that is frequently observed in preterm infants. In turn, decreased blood flow to the brain may result in hypoxic-ischemic brain damage triggering neurodevelopmental abnormalities [106]. Genetic variants in *EBF1* could potentially contribute to low baseline cardiac vagal parasympathetic activity observed in prematurity and autism [49]. Thus, abnormal expression of *EBF1* gene could disrupt the MGVHB by affecting the gut, vagus nerve, the heart, and the brain in prematurity and autism.

*EEFSEC* gene is involved in regulation of selenium incorporation into antioxidant and anti-inflammatory proteins, both of which can affect the timing of birth [9]. Genetic variants in *EEFSEC* have been associated with decreased antioxidant capacity in immature infants [107]. During the full-term pregnancies, the antioxidant defense system is upregulated during the last 6 weeks before birth in preparation for the transition from the hypoxic intrauterine conditions to the hyperoxic extrauterine environment [108] and significantly higher levels of free radicals [107]. On the other hand, premature infants, lacking the critical developmental period, are ill-equipped in antioxidative protection and more at risk of oxidative stress-related brain injury and several pathologies involving an increase in reactive oxygen species (ROS) including autism [109–111]. Oxidative stress damage and brain inflammation [112] as well as inadequate antioxidant capacity have been observed in autism [113]. Furthermore, the effect of *EEFSEC* gene polymorphism may be compounded by the environmentally induced oxidative stress, and existing management practices in preterm infants, including interrupted nursing and the use of antibiotics [114]. Disrupted *EEFSEC* gene expression could thus impact brain development in both prematurity and autism.

*AGTR2* gene encodes angiotensin II receptor type 2 protein that is highly expressed in fetal brain [115] but restricted in the adulthood to brain regions involved in sensory processing [116]. *AGTR2* has been implicated in developmental brain processes [117, 118], neuronal protection, neurite outgrowth, modulation of neuronal excitability, and connectivity [112]. It is also associated with decreased parasympathetic activity observed in both prematurity and autism [47].

Importantly, *AGTR2 receptor subtype*s are involved in the negative regulation of AVP by suppressing baseline systemic AVP levels [119] but in the positive regulation of systemic OXT by increasing the circulating OXT levels [120]. *AGTR2* may thus have a profound impact on the OXT-AVP pathways; abnormalities in the OXT-AVP pathway associated with ASD have been previously reported [121]. AGTR2 is also involved in the regulation of circulation between the placenta and uterus [9].

Genetic variants in *AGTR2* observed in prematurity have also been identified in human X-linked intellectual disability, with clinical features including seizures and autistic behavior [118, 122] and syndromic autism [123] and could significantly disrupt the developing MGVHB axis at several levels.

*WNT4* gene, a member of Wnt signaling pathway involved in the development of CNS, affects cell proliferation, differentiation, cell migration, axon guidance, and synapse formation in CNS. Wnt4 is involved in the retinoic acid-dependent neuronal differentiation [124] and has been implicated in the activity of the gut-brain axis [125]. Importantly, an abnormal synapse formation in neurodevelopmental disorders including autism is attributed to the dysregulation of WNT4 during development [126, 127].

*ADCY5* gene is associated with birth weight [9]; its dysregulation may contribute to a low weight of premature infants. Importantly, disruption of *ADCY5* gene in the dorsal striatum of mouse brain produces autistic-like behaviors [128].

*RAP2C* gene, a member of the Ras GTPase superfamily, functions as a molecular switch of cellular proliferation, differentiation, and apoptosis*. RAP2C* has been associated with preterm delivery [9]. *RAP2C* is involved in GI development and the regulation of selective permeability of the intestinal epithelial barrier [129]. Its dysregulation may contribute to intestinal inflammatory diseases of newborns and children, such as IBD [130] and NEC [131]. Recent studies involving duodenal biopsy speciments derived from ASD patients have shown decreased expression of claudins, proteins involved in intestinal barrier-forming tight junctions (TJ) [37], consistent with the observations of “leaky gut” in autism.

## Discussion

We have previously addressed the abnormalities in the gut-brain axis in autism [20, 32] and prematurity [114]. The present review aimed to examine possible association between prematurity and autism suggested by the epidemiological data. It presents data compiled from clinical and animal studies, comparing symptoms, physiological abnormalities, current mechanistic theories, and genetic advances in support of such association.

The key symptoms that emerged linking prematurity and autism are cognitive and behavioral delays and increased prevalence of gastrointestinal problems (Table 1). Additionally, cardiovascular abnormalities that may be related to parasympathetic dysregulation [64] are observed in both preterm and autistic children.

**Table 1:** Key symptoms, physiological and brain abnormalities linking prematurity and autism

	Prematurity	Autism
Symptoms		
Gastrointestinal	High prevalence of NEC, IBD (28, 29)	High incidence of NEC, IBD (12′ 28, 31) constipation, diarrhea, vomiting, abdominal pain, vomiting
Cardiovascular	Cardiovascular symptoms (48) Heart failure (58) PDA (65)	Cardiovascular symptoms (49) Abnormal cardiac activity (59, 60)
Behavioral/Cognitive	Cognitive and behavioral delays (73) Deficit in social behavior (6)	Cognitive and behavioral delays (74) Deficit in social behavior (75)
Physiological/brain abnormalities		
Gut	“Leaky gut” (38, 44, 45)	“Leaky gut” (19, 20, 32)
Microbiota	Decreased biodiversity and beneficial bacterial species (39)	Decreased biodiversity and beneficial bacterial species (20, 32)
Vagus nerve	Low VNA (48)	Low VNA (49)
Heart	Less complex pattern of HRV (61)	Less complex pattern of HRV (62)
Brain	Reduced volume of temporal, occipital, insular, limbic regions (78) Reduced thalamocortical connectivity (82) IVH (83, 84) PVL (87) Cerebellar injury (87) Damage to CB vermis (73) Reduction in Purkinje cell number (?) Decreased Purkinje cell activity (73) Disrupted connectivity in left hemisphere (96)	Reduced volume of temporal, occipital, insular, limbic regions (79) Abnormal thalamocortical connectivity (81) IVH (85) Cerebellar abnormalities (5, 6); damage to vermis (143) Reduction in Purkinje cell number (93) Decreased Purkinje cell activity (94) Disrupted connectivity in left Hemisphere (98)

These common symptoms were related to the underlying physiological abnormalities that include the “leaky gut,” dysbiosis characterized by decreased biodiversity and increase in pathogenic species, lower vagal activity and decreased parasympathetic activity, abnormal heart activity reflected in the less complex HRV, and regional brain abnormalities related to cognitive and social delays and critical changes in the cerebellum (summarized in Table 1). The common symptoms and underlying physiological abnormalities, in turn, focused our review on the possible disruption of the MGVHB and its emerging role in the development of neuropsychiatric disorders [24].

**Fig. 1:**
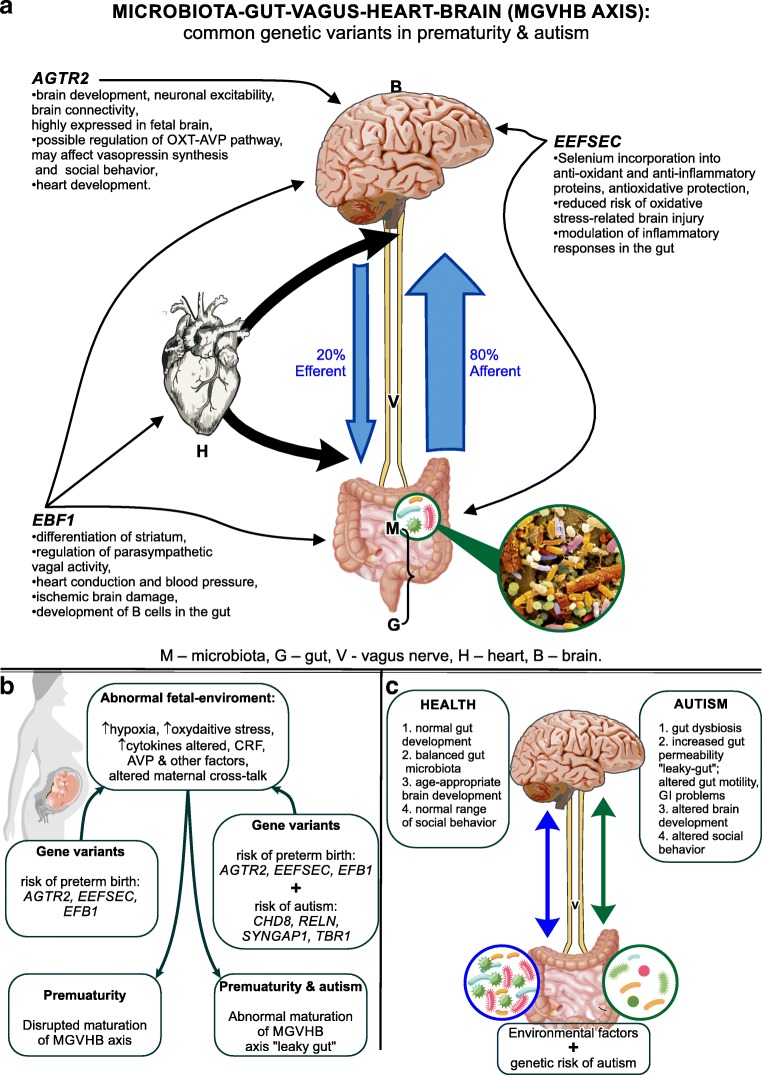
Common genetic variants link the abnormalities in the gut-brain axis in prematurity and autism. **a** Common genetic variants associated with risk of preterm birth and autism: their function and possible site of action in the MGVBH axis. **b** Proposed mechanisms involved in prematurity vs. autism, showing genetic variants associated with risk of preterm birth AGTR2, EERSEC, EB1, and genetic variants of very high risk of autism [109]. **c** Autism as a result of the interaction between disrupted MGVHB axis and the genetic risk factors

Critically, many of the physiological abnormalities observed in the MGVHB axis appear to be regulated by recently identified prematurity risk genes and specifically the genes associated with gestational duration and the risk of preterm birth: *EBF1*, *EEFSEC*, and *AGTR2* [9; Fig. 1a] that are also associated with risk of autism [27]. Thus, based on the clinical, physiological mechanistic, and genetic data, we present a new hypothesis that common genetic variants link the abnormalities in the MGVHB axis in prematurity and autism. The key focal points of the MGVHB axis that may be affected by the gene variants are discussed in the “A Genetic Link Between Prematurity and Autism?” section and summarized below: *EBF1* promotes differentiation of several neuronal cell types including the Purkinje cells [98] whose deficit has been implicated in autistic pathology [93] and has been also reported in premature infants [73]. A variant of *EBF1*, related to the regulation of cardiac conduction and blood pressure, could potentially contribute to decreased blood flow to the brain contributing to hypoxic-ischemic brain damage and neurodevelopmental abnormalities [106]. Genetic variants in *EEFSEC* have been associated with decreased antioxidant capacity in premature infants [107] and could affect the protection against oxidative stress brain damage implicated in prematurity [111] and autism [109–113]. Importantly, coexpression of *EBF1* and *EEFSEC* variants could have additive damaging effect during neurodevelopment. *AGTR2* gene variants have been implicated in several neuronal brain processes [115, 117, 118] and the regulation of OXT and AVP plasma levels [120]; the OXT-AVP pathways have been implicated in autistic pathology [121].

The coexpression of common genetic variants associated with risk of preterm birth and those associated with very high risk of autism [27], such as chromodomain-helicase-DNA-binding protein 8 (CHD8), reelin (RELN), Synaptic Ras GTPase-activating protein 1 (SYNGAP1), and T-box family transcription factor 1 (TBR1), could explain higher risk of autism and/or autistic symptoms in premature infants (Fig. 1b, c); independent expression of high-risk autism gene variants alone could explain why not all autistic children are born prematurely.

The data discussed above support the notion that prematurity and autism share abnormalities in MGVHB axis that may be linked by common genes regulating fetal development. This concept is supported by the fMRI showing diminished functional connectivity in the left hemisphere of the fetal brain, which precedes the preterm birth [96], suggesting that the brain changes in prematurity occur during pregnancy rather than being triggered by the early birth. Many of these changes and specifically damage to the cerebellar structure occur as a result of IVH [83–85] and cerebellar hemorrhagic injury [6] that could be related to cardiovascular abnormalities. This raises an important question whether, perhaps, the cardiovascular changes precede some of the brain changes relevant to the social behavioral deficits in autism and prematurity? It can be speculated that the initial defect in both premature infants and autism would involve the cardiovascular system that in turn would affect the brain development. Such the supposition is supported by observations that prematurity is associated with higher risk of heart failure [58] and an association between abnormal cardiac activity and autism [60]. Furthermore, the congenital heart diseases are associated with increased risk of the neurodevelopmental disabilities including autism [129]. Importantly, *AGTR2* is in the regulation of circulation between placenta and uterus [9] that is sensitive to various vasoactivators, such as angiotensin II and OXT. Importantly, *AGTR2* is involved in the regulation of blood levels of AVP and OXT [119], both related to social behavior [119]. Furthermore, AVP has been implicated in male vulnerability to autism and higher mortality rates in premature male infants [131]. Altered blood levels of OXT have been reported in children with ASD [132] and premature infants [133], underscoring the importance of OXT-AVP pathways in both prematurity and autism.

## Conclusions

Clinical, physiological, and behavioral data support the link between the abnormalities in the MGVHB axis in prematurity and autism, suggested by the epidemiological data. This link is further supported by the identification of common genetic variants involved in MGVHB abnormalities in prematurity and autism. Future studies should aim at identification of additional genes involved in the regulation of the time of birth as well as the reexamination of the autism susceptibility genes in the context of their function in the of MGVHB axis. We tentatively predict that *AGTR2* gene variants and their effects on the OXT-AVP pathways involved in major physiological processes, learning, and social behavior may contribute to further understanding of the link between prematurity and autism. The important outcome of such an association will allow for cross-application of therapies between premature and autistic population. Of special relevance and significance would be an application of probiotic and microbial transfer therapies.
